# Valorization of shrimp waste meal through organic acid treatment: Impact on broilers performance, serum metabolites, digestibility, meat quality, and economic feasibility

**DOI:** 10.1016/j.psj.2026.107858

**Published:** 2026-09-28

**Authors:** Md. Aliar Rahman, Shathi Akter, Jesmin Aktar, Khan Md. Shaiful Islam, Mohammad Al-Mamun, Md. Abul Hashem, Rakhi Chowdhury

**Affiliations:** a Department of Animal Nutrition, Faculty of Animal Husbandry, Bangladesh Agricultural University, Mymensingh, 2202, Bangladesh; b Section of Animal Nutrition and Feed Technology, Department of Animal Sciences, Faculty of Veterinary Medicine and Animal Science, Habiganj Agricultural University, Habiganj, 3300, Bangladesh; c Department of Animal Science, Faculty of Animal Husbandry, Bangladesh Agricultural University, Mymensingh, 2202, Bangladesh

**Keywords:** Shrimp waste meal, Broiler, Formic acid, Profitability, Meat redness

## Abstract

Shrimp waste meal (SWM), is a protein rich by-product whose utilization is limited by high-chitin and ash content. Organic acid-treatment may improve its nutritive value, allowing higher inclusion in broiler diets. Therefore, present study evaluated the effects of citric- and formic acid-treated SWM on growth, digestibility, serum metabolites, meat quality, and profitability in broilers over 35-day. For trial-1, 240 Cobb-500 chicks were allocated to four groups: control, corn-soybean meal (SBM) without SWM; untreated SWM (USWM), citric acid-treated SWM (CSWM), and formic acid-treated SWM (FSWM), with 25% SBM replaced by untreated or treated SWM. For trial-2, 300 Cobb-500 chicks were assigned to five groups: FSWM0 (control) without FSWM, and FSWM25, FSWM50, FSWM75, and FSWM100, where SBM was replaced with 25%, 50%, 75%, and 100% FSWM, respectively. In trial-1, body weight gain and feed conversion ratio in the FSWM group were comparable with the control, whereas USWM and CSWM resulted in poorer performance (*P* < 0.05). The CSWM and FSWM groups had lower abdominal fat and serum triglycerides concentrations than the control and USWM groups *(P* < 0.001). FSWM displayed higher dry matter, nitrogen, and chitin digestibility than USWM and CSWM (*P* < 0.05). Meat redness was optimum in USWM, followed by FSWM and CSWM (*P* < 0.05). In trial-2: performance declined in the FSWM75 and FSWM100 groups, with no variation in the control, FSWM25, and FSWM50 groups. Serum triglycerides decreased linearly with increasing FSWM inclusion (*P* = 0.006). Nitrogen and ash retention remained unchanged in the control, FSWM25, and FSWM50 groups but decreased in FSWM75 and FSWM100, alongside a decline (*P* = 0.001) in chitin digestibility. Meat redness increased in FSWM groups compared with the control (*P* < 0.05). The benefit-cost ratio was similar among the control, FSWM25, and FSWM50 groups but decreased in FSWM75 and FSWM100. Therefore, formic acid-treated SWM outperformed untreated and citric acid-treated SWM in broiler diets. Formic acid-treated SWM can replace 50% SBM, maintaining growth, digestibility, and profitability, while improving meat quality.

## Introduction

Driven by advances in production technologies and rising consumer demand, the poultry industry has expanded rapidly; however, feed costs remain a major constraint, accounting for approximately 60–70% of total production expenses (Buccaro et al., 2023). These costs are highly sensitive to fluctuations in raw material prices, market competition, and the increasing demand for sustainable feed resources. Soybean meal (**SBM**), the predominant plant-based protein source in poultry diets, is costly, import-dependent, and subject to considerable price volatility. Although SBM contains high crude protein levels (44–49% on dry matter) and remains the dominant protein ingredient (Buccaro et al., 2023), its unstable pricing has intensified the search for alternative protein sources to reduce reliance on SBM and stabilize feed costs. Within a circular bio-economy framework, the valorization of agro-industrial by-products like shrimp waste meal (**SWM**) as a feed ingredient represents a promising strategy to enhance resource efficiency, reduce waste, environmental pollution, and lower feed costs without compromising animal performance. In Bangladesh, approximately 23,000 tons of SWM are generated annually, yet remain underutilized as protein source (Islam and Peñarubia, 2021).

Shrimp processing by-products, mainly comprising heads, shells, and exoskeletons, can be converted into SWM with a high crude protein content of up to 52%, particularly when shrimp heads are included (Gernat, 2001). However, the practical use of SWM in poultry diets is limited by its high ash content (approximately 20%) and the presence of chitin, an anti-nutritional polysaccharide accounting for about 14% of the meal (Gernat, 2001; Rahman and Koh, 2014). Chitin can form complexes with dietary proteins and lipids, reducing their accessibility to digestive enzymes and impairing nutrient digestibility and absorption. As a result, excessive inclusion of SWM may compromise growth performance in broiler chickens. Although previous studies have reported that low dietary inclusion levels (2–5%) of SWM in whole diet do not adversely affect broiler performance (Rahman and Koh, 2016a; Onbaşılar et al., 2024), such marginal inclusion limits its economic and practical value as an alternative protein source. In addition to serving as a protein source, SWM provides astaxanthin, a bioactive carotenoid with strong antioxidant properties that may enhance bird health and influence meat pigmentation (Belandria and Morillo, 2013; Jeong and Kim, 2014).

Therefore, processing strategies that reduce chitin content and enhance nutrient availability are essential to improve the feeding value of SWM and enable its greater inclusion in poultry diets. Chemical (hydrochloric acid, formic acid, and sodium hydroxide), biological (*Lactobacillus* spp. and *Saccharomyces cerevisiae*), and enzymatic (chitinase) treatments have been shown to partially improve the nutritional value and digestibility of SWM by reducing ash and chitin contents, thereby allowing moderate dietary inclusion levels (5–15%) in broiler diets (Rahman and Koh, 2016c; Abun et al., 2021; Sangkaew and Koh, 2021). Among these approaches, organic acid treatment, particularly with 3% formic acid, has shown potential for improving the nutritional quality of SWM (Rahman and Koh, 2016c); however, information remains limited regarding the effects of higher acid concentrations and the comparative efficacy of citric and formic acids. Therefore, 5% citric acid and 5% formic acid were selected experimentally to determine whether a higher acid concentration could further improve the nutritional value of SWM. The selection of 5% citric acid was additionally supported by the reported dietary safety margin of up to 6% citric acid in broilers (Islam, 2012). The same concentration was used for both acids to facilitate a direct comparison under a common treatment condition; however, 5% was not assumed to represent an optimum concentration. For the feeding trial, 25% of the SBM was replaced with untreated or acid-treated SWM as a conservative preliminary replacement level. This level was selected with reference to Rahman and Koh (2016c), in which 3% formic acid-treated shrimp meal was included at 5% of the diet and corresponded to an approximately 30% reduction in SBM relative to the control diet, based on the dietary formulations reported in their study. Thus, the 25% SBM replacement used in the present study represented a slightly lower and conservative level for initially evaluating the potential of acid-treated SWM as an SBM substitute. It was hypothesized that treatment with 5% citric acid or formic acid would reduce ash and chitin contents and improve the nutritional value and digestibility of SWM, thereby facilitating its greater use as a partial replacement for SBM in broiler diets.

Accordingly, the present study was designed with two primary objectives: (i) to compare untreated SWM, citric acid–treated SWM, and formic acid–treated SWM based on their nutritional profiles and their effects at a 25% replacement level of SBM on broiler growth performance, serum metabolites, nutrient digestibility, and meat quality in broilers; and (ii) to further evaluate the formic acid–treated SWM, identified as the most promising treatment, at graded levels of SBM replacement (0, 25, 50, 75, and 100%) for its effects on growth performance, serum metabolites, nutrient digestibility, meat quality, and economic feasibility in broiler production.

## Materials and methods

### Collection and processing of shrimp waste

Shrimp waste from black tiger shrimp (*Penaeus monodon*) was obtained from five commercial shrimp processing plants in Chittagong, a major fisheries and fish landing center in southeastern Bangladesh. The waste was manually inspected to remove extraneous materials and minimize contamination, followed by sun-drying. For trial-1, shrimp waste was processed into three forms: untreated shrimp waste, and citric acid-treated shrimp waste, and formic acid-treated shrimp waste. A portion of the untreated shrimp waste was ground and stored as untreated shrimp waste meal (**USWM**) for chemical composition analysis. The remaining portion was treated with either citric acid or formic acid following Rahman and Chowdhury (2025). Briefly, 100 g of shrimp waste was suspended in 300 mL of 5% citric or formic acid solution and incubated at room temperature for 30 min. The mixture was filtered through cheesecloth, rinsed with distilled water, and the residue was sun-dried to constant weight. The dried material was ground to pass through a 1.0-mm mesh screen, producing citric acid-treated shrimp waste meal (**CSWM**) and formic acid-treated shrimp waste meal (**FSWM**). All samples were stored under airtight conditions until chemical composition analysis and *in vitro* digestibility assessment. The *in vitro* assessment was conducted as a preliminary screening step to determine whether the 5% citric acid or 5% formic acid treatments adversely affected dry matter (**DM**) and crude protein (**CP**) digestibility before *in vivo* evaluation, following the procedure described by Rahman and Koh (2016b). For trial-2, shrimp waste meal (**SWM**) was treated exclusively with formic acid using the same procedure described for trial-1, and the treated samples were subsequently stored until chemical composition analysis and the feeding trial.

### Ethics approval

The experimental methodology for handling, killing, and collecting samples from birds was authorized by the Bangladesh Agricultural University Research System's Animal Care and Use Committee (BAURES/ESRC/AH/43).

### Birds and experimental diets

For trial-1, a total of 240 one-day-old Cobb-500 unsexed broiler chicks were purchased from a commercial hatchery and reared for 35 days. Chicks were randomly allocated to four dietary groups following completely randomized design. There were six replicates per group and ten birds per replicate. Dietary groups were: control, corn-soybean meal (**SBM)** diet without SWM; **USWM**, 25% SBM replaced with USWM; **CSWM**, 25% SBM replaced with CSWM; and **FSWM**, 25% SBM replaced with FSWM. Diets were formulated to meet nutrient requirements as recommended in the Cobb-500 Broiler Nutrition and Supplement (2022) guide (www.cobb-vantress.com; l-054-01-22 EN). Starter diets (days 1–14) contained ∼21.73% CP and 2,951 kcal metabolizable energy (**ME**)/kg, while grower diets (days 15–35) contained ∼20.23% CP and 3,060 kcal ME/kg (Table 1).

**Table 1:** Ingredients and nutritive value of experimental diets (%).

**Ingredients**	**Trial-1**^a^								**Trial-2**^b^									
	Starter diet (1-14 days)				Grower diet (15-35 days)				Starter diet (1-14 days)					Grower diet (15-35 days)				
	**Control**	**USWM**	**CSWM**	**FSWM**	**Control**	**USWM**	**CSWM**	**FSWM**	**FSWM**					**FSWM**				
									**0**	**25**	**50**	**75**	**100**	**0**	**25**	**50**	**75**	**100**
Corn	47.30	46.30	46.30	46.30	48.60	47.30	47.30	47.30	50.80	50.10	47.80	44.00	40.00	52.60	48.80	46.40	44.00	40.30
Soybean meal	40.50	30.40	30.40	30.40	37.10	27.83	27.83	27.83	40.00	30.00	20.00	10.00	0.00	35.00	26.25	17.50	8.75	0.00
SWM	0.00	10.10	10.10	10.10	0.00	9.27	9.27	9.27	0.00	10.00	20.00	30.00	40.00	0.00	8.75	17.50	26.25	35.00
Rice polish	4.00	4.00	4.00	4.00	5.00	5.00	5.00	5.00	1.50	4.00	7.00	11.50	15.00	4.00	9.00	12.00	15.50	19.00
Oil	4.00	5.00	5.00	5.00	5.10	6.40	6.40	6.40	3.50	3.50	3.50	3.50	4.00	4.50	4.50	4.50	4.50	4.80
Limestone	1.30	1.30	1.30	1.30	1.30	1.30	1.30	1.30	1.40	0.20	0.00	0.00	0.00	1.55	0.80	0.80	0.00	0.00
DCP	1.90	1.90	1.90	1.90	1.90	1.90	1.90	1.90	1.80	1.20	0.70	0.00	0.00	1.35	0.90	0.30	0.00	0.00
Lysine	0.10	0.10	0.10	0.10	0.10	0.10	0.10	0.10	0.10	0.10	0.10	0.10	0.10	0.10	0.10	0.10	0.10	0.0
Methionine	0.10	0.10	0.10	0.10	0.10	0.10	0.10	0.10	0.10	0.10	0.10	0.10	0.10	0.10	0.10	0.10	0.10	0.10
Broiler premix^c^	0.30	0.30	0.30	0.30	0.30	0.30	0.30	0.30	0.30	0.30	0.30	0.30	0.30	0.30	0.30	0.30	0.30	0.30
Toxin binder	0.10	0.10	0.10	0.10	0.10	0.10	0.10	0.10	0.10	0.10	0.10	0.10	0.10	0.10	0.10	0.10	0.10	0.10
Salkin	0.10	0.10	0.10	0.10	0.10	0.10	0.10	0.10	0.10	0.10	0.10	0.10	0.10	0.10	0.10	0.10	0.10	0.10
Salt	0.30	0.30	0.30	0.30	0.30	0.30	0.30	0.30	0.30	0.30	0.30	0.30	0.30	0.30	0.30	0.30	0.30	0.30
**Nutritive value (**as fed basis, %)																		
Dry matter	88.32	88.25	88.17	88.45	89.16	89.03	89.25	88.97	88.74	88.90	88.81	88.93	89.02	89.13	88.91	89.16	89.05	89.20
Crude protein	22.15	21.47	21.29	22.02	20.90	19.80	20.01	20.24	22.01	21.90	21.75	21.59	21.35	20.30	20.31	20.16	20.10	19.97
Crude fiber	5.12	7.01	6.53	5.97	4.97	6.24	6.15	5.99	5.13	5.98	6.81	7.65	8.43	4.98	5.78	6.49	7.24	7.94
Ether extract	5.39	6.33	6.34	6.36	6.41	7.57	7.59	7.58	4.93	5.31	5.66	6.08	6.83	5.95	6.37	6.71	7.07	7.65
Ash	6.20	7.49	7.22	7.16	6.16	7.34	7.12	7.02	6.03	5.73	6.37	7.18	8.41	5.80	6.04	6.69	6.89	8.00
Calcium^d^	0.98	1.27	1.22	1.16	0.95	1.26	1.23	1.18	0.96	1.21	1.83	2.50	3.30	0.91	1.27	1.83	2.19	2.90
Available phosphorous^d^	0.44	0.57	0.52	0.47	0.41	0.53	0.48	0.46	0.43	0.42	0.43	0.44	0.55	0.36	0.37	0.38	0.44	0.53
Chitin^d^	0.00	1.64	1.59	1.43	0.00	1.52	1.47	1.32	0.00	1.43	2.85	4.28	5.71	0.00	1.25	2.50	3.75	5.00
ME (kcal/kg)^d^	2930	2962	2947	2968	3042	3059	3067	3073	2950	2970	2970	2940	2930	3058	3059	3046	3048	3029

a **:** in trial-1, Control: corn-soybean meal (SBM) diet without shrimp waste meal in starter and grower diet; SWM: shrimp waste meal; USWM: 25% SBM replaced with untreated SWM in starter and grower diet; CSWM: 25% SBM replaced with citric acid-treated SWM in starter and grower diet; and FSWM: 25% SBM replaced with formic acid-treated SWM in starter and grower diet.

b in trail-2, FSWM0: basal corn-SBM diet without formic acid-treated SWM in starter and grower diet; FSWM25: 25% SBM replaced with formic acid-treated SWM in starter and grower diet; FSWM50: 50% SBM replaced with formic acid-treated SWM in starter and grower diet; FSWM75: 75% SBM replaced with formic acid-treated SWM in starter and grower diet; FSWM100: 100% SBM replaced with formic acid-treated SWM in starter and grower diet.

c Siddik et al. (2026).

d Calculated value.

For trial-2, 300 one-day-old Cobb-500 unsexed chicks were reared for 35 days and randomly (following completely randomized design) assigned to five dietary groups with six replicates per group and ten birds per replicate. A corn–SBM diet without FSWM served as the control (**FSWM0**) group. In the remaining groups, SBM was progressively replaced with FSWM at 25%, 50%, 75%, and 100%, designated as **FSWM25, FSWM50, FSWM75**, and **FSWM100**, respectively. Diets were formulated according to Cobb-500 Broiler Nutrition and Supplement (2022) guide (www.cobb-vantress.com; l-054-01-22 EN). Birds were offered hand-mixed starter diets from days 1–14 contained ∼21.72% CP and 2,952 kcal ME/kg, followed by grower diets from days 15–35 contained ∼20.17% CP and 3,048 kcal ME/kg (Table 1).

### Bird’s management, sampling, and analytical procedure

In both trials, birds were housed in well-equipped cages with clean feeders and drinkers and provided *ad libitum* access to mash feed and fresh water. Standard biosecurity measures were enforced, and brooding, lighting, litter management, and vaccination schedules were maintained in accordance with Rahman et al. (2022). Birds were weighed upon arrival and weekly thereafter, while feed intake (**FI**), health status, and mortality were recorded regularly. Body weight (**BW**) gain was determined as the difference between the final and initial live weights. Feed conversion ratio (**FCR**) was calculated by dividing the total FI by the total BW, representing the unit of feed required per unit of weight gain. Nutrient digestibility was determined using chromium oxide (**Cr₂O₃**) as an indigestible marker, from days 29 to 34. During this period, Cr₂O₃ was included at 0.50% of the diet and thoroughly mixed with the mash feed. From days 31 to 33, a polythene sheet was placed over the bedding in each cage immediately after morning feeding to prevent fecal contamination and facilitate collection. Within one hour, approximately 100 g of fecal samples were collected from each cage and pooled. From the pooled sample, 5–10 g was used for daily DM determination, while approximately 20 g was stored at −20°C. Subsequently, samples were thawed, homogenized, pooled across days, dried, ground, and analyzed for chemical composition.

At 36 days of age, two birds per replicate (a total of twelve birds per treatment in both trials) were sacrificed using manual cervical dislocation. Then, blood samples were collected immediately, centrifuged at 3,410 × g for 15 min using centrifuge machine (Hermle Z 306, Germany), and serum samples were collected into Eppendorf tubes and stored at −20°C for biochemical analysis (Akter et al., 2024). After complete bleeding, birds were skinned, visceral organs removed, and weights of dressed carcass and abdominal fat were recorded and expressed as percentages. Breast and thigh meat samples were collected and refrigerated at 4°C for 24 h, then thawed, cleaned, and analyzed for color characteristics namely L*, a*, b* indicated the lightness, redness, and yellowness, respectively using a CR-410 Minolta colorimeter (Japan).

Samples of SWM (untreated, citric-, or formic acid-treated) were analyzed for DM, CP, crude fiber (**CF**), ash, and chitin; experimental diets were analyzed for DM, CP, CF, ether extract, ash, and acid-insoluble ash (**Cr₂O₃**); and excreta were analyzed for DM, CP, ash, chitin, and acid-insoluble ash. These above proximate components were analyzed according to AOAC (2005) standard methods. Chitin content of untreated, citric-, or formic-acid treated SWM was measured as described by Ghanem et al. (2003). The acid-insoluble ash content of the diets and feces was determined according to the method described by Vogtmann et al. (1975). Serum metabolites like triglycerides, total cholesterol (**TC**), and high density lipoprotein (**HDL**) cholesterol were quantified using Urit-810 bio-analyzer with commercial assay kits (HUMAN GmbH, Germany) following the manufacturer’s instructions and the procedures described by Rahman et al. (2026). In addition, the low density lipoprotein (**LDL**) cholesterol was calculated in accordance with Rahman et al. (2024).

### Calculation and statistical analysis

Diets especially control diet ingredients used in both trials were mainly plant-based, their chitin content was assumed to be negligible or tended to zero, consistent with previous findings (Khempaka et al., 2006b; Rahman and Koh, 2016c). Chitin content in the rest experimental diets (containing untreated or treated SWM) was calculated based on an as-fed basis as following formula:

$$\text{Chitin}\;\left(\%\right)=\;\frac{\text{SWM}\;\text{inclusion}\;\text{level}\;\text{in}\;\text{diet}\;\times \;\text{SWM}\;\text{chitin}\;\%\;\left(\text{on}\;\text{DM}\right)\times \;\text{Diet}\;\text{DM}\;\%}{100\;\times \;100}$$

Following the chemical analysis of the diets and feces, the apparent digestibility of DM, chitin, and nitrogen and the retention of ash were determined using the marker-ratio equation as detailed in previous article (Saied et al., 2026). Economic impact of replacing SBM with SWM (trial-2) was evaluated using the benefit-cost ratio (**BCR**) as described by Chowdhury et al. (2024). Total production cost included the market prices of day-old chicks, SWM, formic acid, feed ingredients, and associated management expenses, while total return was based on the market value of live birds at sale. BCR was calculated as net profit divided by total production cost, with values > 1 indicating profitability and values < 1 indicating a loss.

Data from both trials were compiled in Microsoft Excel and analyzed using IBM SPSS (Version 23.0, IBM Corp., USA). One-way ANOVA was used separately to test for dietary groups effects for each trial. Additionally, in trial- 2, orthogonal polynomial contrasts were applied to the data to evaluate the linear, quadratic, and cubic effects of increasing levels of FSWM supplementation in the basal diet on each measured parameters (Redoy et al., 2021). The significant differences among means were determined using Duncan Multiple Range Test’s at *P* < 0.05. Results are presented as mean ± standard deviation.

## Results

### Nutritive value of untreated and organic acid-treated shrimp waste meal

Although no statistical analysis was conducted, the chemical composition and *in vitro* digestibility data (Table 2) suggest that organic acid treatment improved the nutritional profile of SWM. As the results were presented solely as mean (n = 5) values, these findings are interpreted as descriptive rather than inferential. CP content was increased by approximately 5.5% in CSWM and 18.5% in FSWM, compared with untreated SWM. In contrast, CF (9% in CSWM and 17% in FSWM), ash (13% in CSWM and 18% in FSWM), and chitin (3% in CSWM and 13% in FSWM) contents were decreased following organic acid treatments compared with USWM. Notably, the reductions in CF, ash, and chitin content were consistently greater in FSWM than in CSWM. These compositional changes were accompanied by numerically higher *in vitro* DM and CP digestibility in FSWM than in CSWM. Overall, these preliminary findings suggested improved nutritive value and potential nutrient availability following organic acid treatment, particularly with formic acid, and supported the subsequent *in vivo* evaluation of the untreated and acid-treated SWM preparations in broilers.

**Table 2:** Chemical composition and *in-vitro* digestibility of untreated and organic acid-treated shrimp waste meal.

**Components (%)**	**Untreated shrimp waste meal (USWM)**	**Organic acid-treated shrimp waste meal**^a^	
		CSWM	FSWM
Crude protein	37.16	39.20	44.02
Crude fiber	18.29	16.58	15.17
Ash	21.34	18.40	17.48
Chitin	18.41	17.82	16.03
***In-vitro* digestibility (%)**			
Dry matter	68.90	70.19	74.20
Crude protein	67.81	69.01	73.16

a CSWM: 5% citric acid-treated shrimp waste meal; FSWM: 5% formic acid-treated shrimp waste meal.

### Performance parameters

Although the initial BW was similar across all groups, final BW differed significantly (*P* < 0.05; trial-1; Table 3). Final BW and BW gain were highest in the control group (1708 g and 1658 g, respectively), while birds fed the FSWM diet showed no significant differences from the control group for these variables (1668 g and 1620 g, respectively). However, final BW and BW gain in birds fed CSWM were higher than those in the USWM group, and comparable to those in the FSWM group, but significantly lower than those in control group. The lowest final BW and BW gain were recorded in the USWM group (*P* = 0.002). FI was markedly reduced (*P* < 0.05) in the USWM group, approximately 8.6% lower than the control, whereas FI was statistically similar among the birds those fed control, CSWM, and FSWM diets. FCR was found worse in the USWM (2.15) and CSWM (2.14) groups, whereas control (2.05) and FSWM (2.07) groups exhibited statistically similar FCR values. Abdominal fat % was markedly reduced in birds fed organic acid-treated SWM, with CSWM and FSWM groups showing approximately 16% and 20% lower abdominal fat, respectively, compared with the control.

**Table 3:** Influences of experimental diets on growth performance and carcass quality in broilers (trial-1)a.

**Variables**	**Control**	**USWM**	**CSWM**	**FSWM**	**SEM**	***P*-value**
Initial BW, g	49.24±0.92	48.10±1.35	47.05±0.92	47.97±0.74	0.341	0.145
Final BW, g	1708^a^±25.53	1511^c^±39.21	1602^b^±18.58	1668^ab^±63.15	24.53	0.002
BW gain, g	1658^a^±26.44	1463^c^±38.13	1555^b^±17.75	1620^ab^±63.75	24.43	0.002
Feed intake, g	3436^a^±34.27	3140^b^±83.05	3324^a^±71.30	3325^a^±75.02	36.23	0.005
FCR	2.07^a^±0.03	2.15^b^±0.01	2.14^b^±0.02	2.05^a^±0.04	0.014	0.006
Abdominal fat, %	1.41^a^±0.04	1.34^ab^±0.18	1.18^bc^±0.04	1.13^c^±0.10	0.058	0.034

a Control: corn-soybean meal (SBM) diet without shrimp waste meal (SWM); USWM: 25% SBM replaced with untreated SWM; CSWM: 25% SBM replaced with citric acid-treated SWM; FSWM: 25% SBM replaced with formic acid-treated SWM; BW: body weight; FCR: feed conversion ratio; g: gram; %: percentage; SEM: standard error mean; Value represents mean ± standard deviation ^a-c^ Mean values within the same row followed by different superscripts are significantly different at the *P* < 0.05 level.

In trial-2, higher inclusion levels of FSWM, particularly FSWM75 and FSWM100, appeared to negatively affect final BW, BW gain, and FI in broilers (Table 4). Although final BW, BW gain, and FI did not differ significantly among FSWM0, FSWM25, and FSWM50 groups, a declining (linear and quadratic; *P* < 0.001) trend was observed from FSWM50 group to forward. FCR was best as lowest in the FSWM0 group and worse as highest in FSWM100 group. FCR value in FSWM25 (1.73) and FSWM50 (1.76) was statically comparable with FSWM0 (1.70), whereas FSWM75 (1.88) and FSWM100 (1.91) exhibited significantly higher FCR than all other groups (linear; *P* < 0.001), indicating reduced feed efficiency at the higher inclusion levels. Supplementation of FSWM significantly reduced abdominal fat deposition compared with the control (*P* < 0.001), and this response was stable across all FSWM inclusion levels, however no detectable differences was found among the FSWM groups.

**Table 4:** Effects of different level of formic acid-treated shrimp waste meal on performance and abdominal fat content in broilers (trial-2)a.

**Variables**	**FSWM**					***P*-value**			
	**0**	**25**	**50**	**75**	**100**	**OWA**	**L**	**Q**	**C**
Initial BW, g	43.86±0.46	43.41±0.54	43.50±1.26	43.07±1.02	43.33±0.97	0.870	0.413	0.655	0.934
Final BW, g	1915.67^a^±40.08	1880.67^a^±28.38	1843.00^a^±51.00	1616.33^b^±41.49	1358.33^c^±19.86	<0.001	<0.001	<0.001	0.686
BW gain, g	1871.81^a^±40.07	1837.26^a^±27.95	1799.50^a^±49.87	1573.26^b^±42.48	1315.01^c^±20.66	<0.001	<0.001	<0.001	0.684
FI, g	3185.50^a^±99.71	3173.21^ab^±103	3163.83^ab^±79.67	2957.75^b^±71.70	2513.83^c^±54.02	<0.001	<0.001	<0.001	0.145
FCR	1.70^b^±0.02	1.73^b^±0.03	1.76^b^±0.04	1.88^a^±0.01	1.91^a^±0.04	<0.001	<0.001	0.103	0.136
Abd. fat, %	1.50^a^±0.03	1.30^b^±0.04	1.29^b^±0.03	1.25^b^±0.02	1.24^b^±0.03	<0.001	<0.001	<0.001	0.019

a FSWM0 (control): corn-soybean meal (SBM) diet without formic acid-treated shrimp waste meal (SWM); FSWM25: 25% SBM replaced with formic acid-treated SWM; FSWM50: 50% SBM replaced with formic acid-treated SWM; FSWM75: 75% SBM replaced with formic acid-treated SWM; FSWM100: 100% SBM replaced with formic acid-treated SWM; BW: body weight; FI: feed intake; FCR: feed conversion ratio; g: gram; Abd.: abdominal; %: percentage; OWA: one way ANOVA; L: linear, Q: quadratic; C: cubic; Value represents mean ± standard deviation ^a-c^ Mean values within the same row followed by different superscripts are significantly different at the *P* < 0.05 level.

### Serum metabolites and nutrient digestibility

Inclusion of untreated and organic acid-treated SWM (trial-1) significantly affected serum triglycerides concentrations (Table 5; *P* < 0.05). Birds in the control group exhibited the highest triglycerides level (61.27 mg/dL), whereas concentrations declined progressively in the CSWM and FSWM groups, with the lowest value observed in FSWM (51.43 mg/dL). In contrast, TC, HDL cholesterol, and LDL cholesterol concentrations were not influenced by dietary untreated or treated SWM (*P* > 0.05). In trial-2, dietary inclusion of FSWM did not affect serum TC, HDL cholesterol, and LDL cholesterol across all groups (Table 6; *P* > 0.05). Triglycerides concentrations were significantly (*P* = 0.001) reduced in all FSWM supplemented groups compared with the control (FSWM0) group, whereas dietary groups within the FSWM supplemented groups (FSWM25, FSWM50, FSWM75, and FSWM100) exhibited a significant linear (*P* = 0.006) response.

**Table 5:** Influences of experimental diets on serum metabolites and digestibility in broilers (trial-1)a.

**Variables**	**Control**	**USWM**	**CSWM**	**FSWM**	**SEM**	***P*-value**
**Serum metabolites (mg/dL)**						
Triglycerides	61.27^a^±1.95	63.54^a^±1.17	55.11^b^±1.63	51.43^b^±1.85	1.509	<0.001
Total cholesterol	81.73±2.97	82.60±1.32	81.33±1.51	80.98±3.30	0.985	0.063
HDL cholesterol	54.48±2.64	51.56±2.43	50.79±3.44	50.30±2.88	0.431	0.324
LDL cholesterol	19.13±2.0	20.01±3.41	19.15±2.30	18.10±2.73	0.125	0.942
**Nutrients digestibility (%)**						
Dry matter	73.26^a^±1.06	69.06^b^±0.98	70.07^b^±1.23	72.41^a^±0.74	0.536	0.005
Nitrogen	72.13^a^±1.03	66.18^c^±0.90	69.08^b^±0.89	71.54^a^±0.46	0.596	0.001
Chitin	-	23.67^c^±0.96	31.16^b^±0.96	38.20^a^±0.74	2.812	<0.001
Ash retention	42.15^a^±1.14	34.35^c^±0.61	37.39^b^±1.20	41.98^a^±0.91	0.527	0.005

a Control: corn-soybean meal (SBM) diet without shrimp waste meal (SWM); USWM: 25% SBM replaced with untreated SWM; CSWM: 25% SBM replaced with citric acid-treated SWM; FSWM: 25% SBM replaced with formic acid-treated SWM; HDL: high density lipoprotein; LDL: low density lipoprotein; mg/dL: milligram per deciliter; %: percentage; -: not determined; SEM: standard error mean; Value represents mean ± standard deviation ^a-c^ Mean values within the same row followed by different superscripts are significantly different at the *P* < 0.05 level.

**Table 6:** Influences of different level of formic acid-treated shrimp waste meal on serum metabolites and digestibility in broilers (trial-2)a.

**Variables**	**FSWM**					***P*-value**			
	**0**	**25**	**50**	**75**	**100**	**OWA**	**L**	**Q**	**C**
**Serum metabolites (mg/dL)**									
Triglycerides	62.42^a^±1.27	60.19^b^±1.03	59.81^b^±0.71	59.33^b^±0.75	58.46^b^±0.95	0.001	0.006	0.235	0.233
Total cholesterol	79.99±0.89	79.79±1.03	80.26±1.34	80.36±1.77	80.08±1.24	0.983	0.758	0.852	0.662
HDL cholesterol	47.19±0.55	45.95±0.83	47.99±1.71	47.91±1.79	47.33±0.59	0.317	0.337	0.770	0.120
LDL cholesterol	20.32±0.38	21.81±1.25	20.31±1.11	20.59±1.63	21.05±1.65	0.595	0.914	0.924	0.206
**Nutrients digestibility (%)**									
Dry matter	74.08^a^±1.63	71.73^ab^±0.48	70.51^b^±1.90	65.04^c^±1.03	63.29^c^±0.69	0.001	0.001	0.290	0.290
Nitrogen	75.21^a^±0.88	73.85^a^±0.77	72.96^a^±1.59	68.67^b^±0.62	62.15^c^±1.04	0.001	0.001	0.001	0.184
Chitin	-	38.20^a^±1.20	35.17^a^±3.59	29.23^b^±0.97	25.37^c^±1.06	0.001	0.001	0.001	0.001
Ash retention	42.92^a^±1.71	41.05^a^±1.01	39.73^ab^±1.51	36.81^bc^±1.24	34.51^c^±0.60	0.001	0.001	0.392	0.973

a FSWM0 (control): corn-soybean meal (SBM) diet without formic acid-treated shrimp waste meal (SWM); FSWM25: 25% SBM replaced with formic acid-treated SWM; FSWM50: 50% SBM replaced with formic acid-treated SWM; FSWM75: 75% SBM replaced with formic acid-treated SWM; FSWM100: 100% SBM replaced with formic acid-treated SWM; HDL: high density lipoprotein; LDL: low density lipoprotein; mg/dL: milligram per deciliter; %: percentage; - = not determined; OWA: one way ANOVA; L: linear, Q: quadratic; C: cubic; Value represents mean ± standard deviation ^a-c^ Mean values within the same row followed by different superscripts are significantly different at the *P* < 0.05 level.

Inclusion of untreated, citric acid, and formic acid-treated SWM (trial-1) significantly (*P* < 0.001) influenced DM and chitin digestibility as well as nitrogen and ash retention in broilers (Table 5). DM digestibility was highest in the control, lowest in the USWM, and followed by the CSWM, whereas the FSWM exhibited values comparable to the control and significantly higher than the USWM and CSWM (*P* = 0.005). Chitin digestibility was highest (*P* < 0.001) in FSWM, followed by CSWM and USWM, highlighting the effectiveness of acid-treatment in enhancing chitin utilization. Nitrogen and ash retention were highest (*P* = 0.001) in the control and comparable in FSWM but reduced in CSWM, and lowest in USWM. The inclusion of FSWM at different levels (trial-2) significantly affected DM and chitin digestibility as well as ash and nitrogen retention (Table 6). DM and chitin digestibility in FSWM25 and FSWM50 remained comparable to the control, but declined (*P* = 0.001) markedly at higher inclusion levels (FSWM75 and FSWM100). Nitrogen and ash retention followed a similar pattern, being lowest (linear and quadratic; *P* = 0.001) in the FSWM100, intermediate in the FSWM75, and comparable (*P* = 0.001) to the control (FSWM0), FSWM25, and FSWM50.

### Meat color

Inclusion of untreated and organic acid-treated SWM in both trials significantly affected the a* value of broiler breast and thigh meat (Table 7, Table 8). In trial-1, birds fed the USWM diet exhibited the highest a* value for both breast (*P* = 0.025) and thigh (*P* = 0.027) meat, whereas the CSWM and FSWM groups showed intermediate values, being lower than USWM but higher than the control. However, experimental diets did not show any noticeable effects on breast and thigh meat L* and b* value (Table 7). In trial-2, a* in both breast and thigh meat increased (linear, *P* = 0.003) with increasing inclusion levels of FSWM. Although no differences were observed among the FSWM supplemented groups, all supplemented groups differed significantly (*P* < 0.05) from the control (FSWM0) group. In addition, dietary formic acid-treated SWM did not influence L* or b* value of breast and thigh meat (Table 8).

**Table 7:** Influences of experimental diets on meat color in broilers (trial-1)a.

**Variables**	**Control**	**USWM**	**CSWM**	**FSWM**	**SEM**	***P*-value**
**Breast meat color**						
Lightness (L*)	60.67±1.18	60.11±1.41	61.53±0.84	59.80±1.32	0.357	0.374
Redness (a*)	4.74^c^±0.53	7.61^a^±1.35	5.54^b^±0.82	5.83^b^±0.95	0.411	0.025
Yellowness (b*)	12.21±0.38	12.67±0.81	11.95±0.81	12.76±1.11	0.225	0.604
**Thigh meat color**						
Lightness (L*)	41.37±3.43	42.56±6.88	39.72±5.84	40.25±6.27	1.455	0.931
Redness (a*)	4.01^c^±0.84	6.70^a^±1.13	5.06^b^±0.76	5.68^b^±0.60	0.362	0.027
Yellowness (b*)	9.79±0.81	10.90±0.83	9.11±0.53	10.56±1.13	0.296	0. 122

a Control: corn-soybean meal (SBM) diet without shrimp waste meal (SWM); USWM: 25% SBM replaced with untreated SWM; CSWM: 25% SBM replaced with citric acid-treated SWM; FSWM: 25% SBM replaced with formic acid-treated SWM; SEM: standard error mean; Value represents mean ± standard deviation ^a-c^ Mean values within the same row followed by different superscripts are significantly different at the *P* < 0.05 level.

**Table 8:** Effects of different level of formic acid-treated shrimp waste meal on meat color in broilers (trial-2)a.

**Variables**	**FSWM**					***P*-value**			
	**0**	**25**	**50**	**75**	**100**	**OWA**	**L**	**Q**	**C**
**Breast meat**									
Lightness (L*)	57.68±1.51	58.22±1.70	58.06±0.97	58.25±1.03	57.76±0.52	0.942	0.801	0.524	0.663
Redness (a*)	4.59^b^±0.35	5.92^a^±0.57	6.20^a^±0.53	6.29^a^±0.68	6.35^a^±0.61	0.011	0.002	0.047	0.240
Yellowness (b*)	10.61±1.07	10.96±0.79	11.16±0.90	9.60±0.41	10.00±0.37	0.129	0.088	0.327	0.151
**Thigh meat**									
Lightness (L*)	39.70±1.54	40.56±1.20	40.39±1.46	40.25±1.03	40.16±1.22	0.965	0.934	0.534	0.993
Redness (a*)	5.71^b^±0.36	7.07^a^±0.61	7.34^a^±0.56	7.37^a^±0.61	7.54^a^±0.51	0.016	0.003	0.048	0.339
Yellowness (b*)	10.61±1.06	10.96±0.77	11.16±0.92	9.60±0.46	10.00±0.27	0.129	0.088	0.327	0.153

a FSWM0 (control): corn-soybean meal (SBM) diet without formic acid-treated shrimp waste meal (SWM); FSWM25: 25% SBM replaced with formic acid-treated SWM; FSWM50: 50% SBM replaced with formic acid-treated SWM; FSWM75: 75% SBM replaced with formic acid-treated SWM; FSWM100: 100% SBM replaced with formic acid-treated SWM; OWA: one way ANOVA; L: linear, Q: quadratic; C: cubic; Value represents mean ± standard deviation ^a-b^ Mean values within the same row followed by different superscripts are significantly different at the *P* < 0.05 level.

### Production cost

The inclusion of FSWM significantly affected production cost and economic efficiency (*P* < 0.001; Table 9). Total feed and production costs decreased with increasing FSWM levels, primarily due to reduced SBM cost, despite a proportional increase in FSWM cost. However, bird selling price also declined with higher inclusion levels, reflecting reduced performance. Consequently, profitability and BCR decreased progressively, with acceptable economic returns observed in the control, FSWM25, and FSWM50 groups, while FSWM75 showed reduced profit and FSWM100 resulted in negative returns. Overall, moderate inclusion (up to 50% of SBM replacement) maintained economic viability, whereas higher levels impaired profitability.

**Table 9:** Effects of different level of formic acid-treated shrimp waste meal on production cost (US Dollar) in broilers (trial-2) a.

**Variables (per bird)**	**FSWM**					***P*-value**			
	**0**	**25**	**50**	**75**	**100**	**OWA**	**L**	**Q**	**C**
Total feed cost	1.426^a^±0.045	1.400^a^±0.045	1.372^a^±0.035	1.264^b^±0.031	1.061^c^±0.021	<0.001	<0.001	<0.001	0.193
SBM cost	0.591^a^±0.019	0.442^b^±0.014	0.294^c^±0.007	0.137^d^±0.003	0.000^e^±0.000	<0.001	<0.001	0.511	0.401
FSWM cost	0.000^e^±0.000	0.135^d^±0.004	0.268^c^±0.007	0.376^b^±0.009	0.417^a^±0.008	<0.001	<0.001	<0.001	<0.001
Total production cost	1.994^a^±0.045	1.968^a^±0.045	1.940^a^±0.035	1.832^b^±0.031	1.629^c^±0.021	<0.001	<0.001	<0.001	0.193
Bird selling price	2.246^a^±0.048	2.205^a^±0.034	2.159^a^±0.060	1.888^b^±0.051	1.578^c^±0.025	<0.001	<0.001	<0.001	0.684
Profit	0.253^a^±0.004	0.237^a^±0.014	0.220^a^±0.043	0.056^b^±0.021	−0.051^c^±0.014	<0.001	<0.001	<0.001	0.201
Benefit-cost ratio	1.268^a^±0.012	1.205^a^±0.097	1.132^a^±0.221	0.303^b^±0.109	−0.314^c^±0.085	<0.001	<0.001	<0.001	0.017

a FSWM0 (control): corn-soybean meal (SBM) diet without formic acid-treated shrimp waste meal (SWM); FSWM25: 25% SBM replaced with formic acid-treated SWM; FSWM50: 50% SBM replaced with formic acid-treated SWM; FSWM75: 75% SBM replaced with formic acid-treated SWM; FSWM100: 100% SBM replaced with formic acid-treated SWM; SBM: soybean meal; FSWM: formic acid-treated shrimp waste meal; OWA: one way ANOVA; L: linear; Q: quadratic; C: cubic; Value represents mean ± standard deviation ^a-e^ Mean values within the same row followed by different superscripts are significantly different at the *P* < 0.05 level; 1 US Dollar = 125 Bangladesh Taka.

## Discussions

The observed improvements in chemical composition and *in vitro* digestibility of SWM following organic acid-treatment are consistent with previous reports on acid-mediated modification of animal by-product as feeds. The increase in CP content in both CSWM and FSWM may be attributed to partial demineralization of the exoskeletal matrix and the solubilization of non-protein components, resulting in a relative enrichment of protein fractions. Similar enhancements in protein availability and nutrient digestibility following organic acid treatment of SWM have been reported by Rahman and Koh (2016c), who attributed these effects to improved nitrogen recovery and disruption of structural barriers limiting enzymatic accessibility.

The reductions in CF, ash, and chitin content further indicate the effectiveness of organic acids in disrupting the chitin-protein-mineral complex characteristic of shrimp shell waste. Chitin, largely indigestible for monogastric animals, is a key limiting factor in the utilization of SWM (Khempaka et al., 2006a, 2006b). The greater reduction observed in FSWM compared with CSWM may indicate more extensive disruption of the mineral-chitin matrix by formic acid, potentially due to its lower molecular weight and higher penetration capacity. This is consistent with the previous findings indicating that organic acids effectively demineralize crustacean shells and reduce residual mineral content, thereby facilitating the removal of structural barriers associated with chitin and ash fractions (El-Araby et al., 2022). These compositional improvements were accompanied by improved *in vitro* DM and CP digestibility, particularly in FSWM. The reduction in chitin and mineral fractions likely enhanced enzyme accessibility to protein substrates. Previous studies have demonstrated a negative relationship between chitin concentration and protein digestibility in shrimp by-product based diets for poultry and aquatic animals (Khempaka et al., 2011; Abun et al., 2022; Eggink et al., 2025). Therefore, the higher digestibility observed in FSWM may reflect more extensive degradation of indigestible components, supporting its potential as an effective processing approach to enhance the nutritional value of SWM in broiler diets.

The comparable initial BW across the dietary groups (trial-1) indicates comparable baseline BW among treatments. Birds fed FSWM achieved significantly higher final BW and BW gain than those receiving USWM and CSWM, and performed comparably to the control diet. These findings suggest that formic acid treatment effectively alleviated some of the nutritional constraints associated with SWM utilization. The inferior performance of birds fed USWM is consistent with previous reports highlighting the limitations of untreated SWM, including high chitin content, low protein digestibility, and mineral imbalance (Khempaka et al., 2011; Abun et al., 2021, 2022). Chitin forms complexes with proteins and lipids, reducing enzymatic accessibility and potentially limiting nutrient utilization, which may have contributed to the reduced final BW, BW gain, and poorer FCR observed in this group. Although FI was similar among treatments except for the significantly lower FI in USWM, the relatively higher absolute FCR values, including that of the control, may partly reflect the experimental conditions, such as the use of mash diets, open-sided housing with ambient temperature fluctuations, and the absence of antibiotic growth promoters or other performance-enhancing additives. These factors may have limited overall growth efficiency without necessarily reducing FI. However, because all treatments were evaluated under the same conditions, the relative differences in FCR among dietary treatments remain valid for comparison.

Beyond these general experimental factors, the lower FI observed in USWM may also be related to the relatively high chitin and ash contents of untreated SWM, which could affect palatability and gastrointestinal function. In addition, the physicochemical properties of dietary fiber may alter the rheological characteristics of intestinal digesta, potentially increasing digesta viscosity at certain inclusion levels. Elevated intestinal viscosity can restrict the diffusion and interaction of digestive enzymes with their substrates and impair nutrient digestion and absorption, thereby reducing nutrient utilization efficiency (Jha and Mishra, 2021). Thus the greater chitin load in SWM added diet may have imposed additional constraints on digestive efficiency, contributing to its lower FI, poorer growth performance, and higher FCR. Nevertheless, because intestinal digesta viscosity was not directly measured in the present study, its contribution should be considered a plausible mechanism rather than a demonstrated causal pathway.

Organic acid treatment markedly appeared to alleviate these nutritional constraints, particularly in the FSWM group. The superior performance of the FSWM group relative to CSWM indicates that formic acid was more effective than citric acid in enhancing SWM utilization. This may be attributed to more extensive disruption of the mineral-chitin matrix and improved nutrient availability following formic acid treatment (Pearlin et al., 2020). Additionally, formic acid exerts well-documented antimicrobial effects in the gastrointestinal tract, reducing pathogenic bacterial load and promoting a more favorable gut environment for nutrient absorption (Dibner and Buttin, 2002; Ricke, 2003). The comparable FI and FCR between the FSWM and control groups further support the improved utilization of formic acid treatment SWM under the conditions of the present study.

A notable reduction in abdominal fat % was observed in birds fed organic acid-treated SWM, particularly in the FSWM group. This effect may be attributed to improved protein utilization and greater partitioning of nutrients toward lean tissue accretion rather than fat deposition. Additionally, organic acids have been reported to influence lipid metabolism through changes in gut microbiota and short chain fatty acids production, potentially affecting lipogenesis (Liu et al., 2021; Jing et al., 2026). The greater reduction in abdominal fat observed with its overall superior performance and may reflect improved nutrient utilization following formic acid treatment.

On the other hand, increasing dietary inclusion levels (trial-2) of FSWM revealed a clear threshold beyond which growth performance deteriorated. While FSWM25 and FSWM50 maintained growth performance and showed feed efficiency comparable to the control (FSWM0) group, higher inclusion levels (FSWM75 and FSWM100) significantly reduced final BW, BW gain, FI, and deteriorated FCR. This dose-dependent decline suggests that, despite acid treatment, excessive SWM inclusion may impose nutritional and physicochemical constraints, including greater chitin and ash loads a potentially less favorable amino acid profile, which may limit nutrient utilization (Rahman and Koh, 2016a). Because the amino acid profile of SWM used in the present study was not directly analyzed, the contribution of amino acid imbalance to these responses remains speculative. As discussed previously, the increasing chitin load may also potentially alter intestinal digesta rheology and viscosity, thereby restricting enzyme–substrate interactions and nutrient diffusion and compromising digestion and absorption. These effects may become increasingly important at higher FSWM inclusion levels and could collectively contribute to the deterioration in growth performance (Ravindran and Blair, 1993; Gernat, 2001; Khempaka et al., 2006a; Jha and Mishra, 2021).

The reduction in FI at higher FSWM inclusion levels further suggests decreased palatability or an increased dietary acid load, both of which can negatively influence voluntary FI when inclusion exceeds optimal thresholds. Moreover, the markedly poorer FCR at the highest inclusion level reflects inefficient nutrient utilization and increased metabolic costs associated with dietary imbalance. These findings emphasize the importance of optimizing inclusion levels of alternative protein sources, even when effective processing methods are applied. Interestingly, no significant differences in abdominal fat % were observed among the FSWM supplemented groups. This may be attributed to the uniform application of formic acid treatment across all diets containing FSWM. Although the inclusion level of FSWM varied, the associated formic acid treatment exposure and thus its metabolic effects remained relatively constant, likely exerting a consistent modulatory influence on lipid metabolism across the dietary groups. This suggests that the observed reduction in abdominal fat is primarily associated with the presence of formic acid rather than the inclusion level of treated SWM. Overall, the present findings demonstrate that formic acid treatment significantly improves the feeding value of SWM, enabling its inclusion at moderate levels without compromising growth performance, or feed efficiency. However, excessive inclusion remains detrimental, underscoring the need for balanced diet formulation and careful optimization of inclusion levels in practical broiler ration.

The present study demonstrate that dietary inclusion of organic acids-treated SWM, selectively modulates lipid metabolism in broiler by reducing serum triglycerides concentrations without affecting TC or their lipoprotein fractions (HDL and LDL). The consistent reduction in triglycerides observed in both trials, particularly in birds fed FSWM, indicates a pronounced hypolipidemic effect of organic acids (Al-Fadhli et al., 2018; Waghmare et al., 2025) treatment. This reduction may be attributed to improved nutrient utilization and altered energy partitioning. Organic acids, particularly formic acid, enhance gut health by suppressing pathogenic bacteria and improving intestinal integrity, thereby increasing nutrient absorption efficiency (Dibner and Buttin, 2002; Ricke, 2003). Improved protein digestibility and amino acid availability may favor lean tissue accretion over lipid deposition, reducing substrate availability for triglycerides synthesis (Leeson and Summers, 2001). Moreover, organic acids may influence gut microbial fermentation, potentially increasing short chain fatty acids such as propionate, which has been associated with suppression of hepatic lipogenesis (den Besten et al., 2013; Li et al., 2021). However, this mechanism was not directly evaluated in the present study. The absence of changes in serum total, HDL, and LDL cholesterol across suggest that cholesterol metabolism in broiler is relatively stable and less responsive to dietary interventions compared with triglycerides metabolism under isoenergetic and isonitrogenous conditions (Leeson and Summers, 2001; Safaa et al., 2014). Moreover, the lack of differences among FSWM inclusion levels indicates that the triglycerides lowering effect was primarily driven acid treatment rather than the inclusion rate.

Organic acid also improved nutrient digestibility and nitrogen retention in broilers. In trial-1, FSWM exhibited higher DM digestibility, ash, and nitrogen retention compared with USWM, along with improved chitin utilization, indicating enhanced nutrient availability. This improvement is likely due to disruption of chitin-protein complexes, increased mineral solubility, and enhanced protein accessibility, as well as favorable modulation of gut microbiota (Dibner and Buttin, 2002; Ricke, 2003). Inclusion of FSWM at 25% and 50% maintained digestibility and nitrogen retention comparable to the FSWM0 (trial-2), whereas higher inclusion levels (75% and 100%) significantly impaired these parameters. This decline is likely associated with residual chitin, increased ash content, and potential amino acid imbalance that exceed the digestive capacity of broiler (Ravindran and Blair, 1993; Khempaka et al., 2011). The progressive reduction in chitin digestibility further indicates a threshold beyond which nutrient utilization becomes compromised. However, the amino acid profile of the SWM used in the present study was not directly measured, which limits interpretation of the amino acid adequacy of the experimental diets and highlights the need for direct amino acid characterization in future studies.

Broiler breast meat color, particularly a*, is an important quality attribute that may influence consumer perception and market value. In the present study, dietary inclusion of untreated or treated SMW significantly affected breast and thigh meat a* in both trials. Dietary components can influence meat color through their effects on muscle pigment concentration, oxidative stability, and myoglobin chemistry. The observed increase in meat a* may be associated with the presence of natural carotenoid pigments, particularly astaxanthin, which may enhance color intensity and contribute to myoglobin stability by reducing oxidative discoloration in muscle tissue. Previous studies have demonstrated that dietary carotenoids, including astaxanthin, can improve meat color and oxidative stability in poultry and other livestock species (Carballo et al., 2019; Hosseindoust et al., 2020; Nabi et al., 2020). Consistent with these findings, deeper egg yolk pigmentation in hens fed graded levels of FSWM further supports the potential role of shrimp derived carotenoids in enhancing product coloration (Rahman and Chowdhury, 2025). Furthermore, improved astaxanthin status may help to maintain myoglobin in its reduced form, thereby enhancing a* without affecting L* or b*, as observed in the present study. Similar findings have been reported in which dietary antioxidant improved meat color stability without altering other color parameters (Velasco and Williams, 2011; Leal, 2019). From a consumer perspective, changes in meat a* may influence visual perception and acceptance, even though broiler meat is generally characterized as white meat. Therefore, the increased a* observed with FSWM inclusion could potentially influence consumer appreciation; however, whether this change would be perceived positively or negatively remains uncertain and may depend on consumer expectations and market preferences. Because consumer sensory acceptance was not evaluated in the present study, further sensory studies are warranted to determine whether the observed change in a* has any practical effect on consumer preference. Moreover, as antioxidant activity and muscle pigment concentrations were not directly measured in this study, the proposed mechanisms should be considered potential explanations rather than confirmed mechanisms underlying the observed changes in meat a*.

The present findings indicate that inclusion of FSWM significantly influences production economics in broiler production. Although total feed and production costs decreased with increasing FSWM levels due to the reduced reliance on SBM, this economic advantage was offset at higher inclusion levels by a decline in bird selling price, reflecting reduced growth performance. As a result, profitability and BCR decreased progressively, with optimal economic returns observed at moderate inclusion levels (up to 50%), while higher inclusion levels (75% and 100%) led to diminished or negative returns. The reduction in production cost with partial replacement of conventional protein sources by alternative feed ingredients has been widely reported and is primarily attributed to lower ingredient costs (Ravindran, 2013). However, the economic efficiency of such substitutions depends not only on feed cost but also on their impact on growth performance and market weight. In the present study, the decline in selling price at higher inclusion levels suggests that the negative effects on growth performance outweighed the savings in feed cost, thereby reducing overall profitability. Similar patterns have been observed in previous studies where increasing inclusion of alternative protein sources improved feed cost but compromised growth performance beyond certain thresholds, leading to reduced economic returns (Vadivel and Pugalenthi, 2010; Hagan et al., 2016). In present study, the decline in BCR at higher inclusion levels highlights the need to balance cost savings with performance efficiency in diet formulation. Moderate inclusion levels that maintain growth performance while partially replacing conventional ingredients yield the most favorable economic returns. These results indicate that although FSWM can reduce feed cost, its inclusion should be optimized to avoid performance-related losses that ultimately reduce profitability.

## Conclusions

The present study demonstrates that organic acid treatment, particularly with formic acid, enhances the nutritional value of SWM by improving its chemical composition and digestibility through reduction of chitin and ash. These improvements translated into superior growth performance, feed efficiency, and nutrient digestibility in broilers, with FSWM showing greater efficacy than untreated SWM and CSWM. Moderate inclusion levels (up to 50%) of FSWM maintained performance and economic returns, reduced abdominal fat and serum triglycerides, and improved meat redness compared with the control. Overall, formic acid treatment represents an effective strategy to valorize shrimp waste as an alternative protein source in broiler diets, provided that inclusion levels are carefully optimized to avoid performance and economic losses.

## Data and model availability statement

None of the data were deposited in an official repository.

## Declaration of generative AI and AI-assisted technologies in the writing process

English artificial intelligence-assisted technologies were not used during the writing process.

## Financial support statement

The authors gratefully acknowledge the Bangladesh Agricultural University Research System (Project ID: BAURES/783(50), BAU) and Ministry of Science and Technology, Bangladesh (Project ID: MoST/BS 39) for providing financial support for research.

## Disclosures

The authors declare the following financial interests/personal relationships which may be considered as potential competing interests:

Rakhi Chowdhury reports financial support was provided by Bangladesh Agricultural University Research System, BAU and Ministry of Science and Technology, Bangladesh. If there are other authors, they declare that they have no known competing financial interests or personal relationships that could have appeared to influence the work reported in this paper.

## Author Contributions

Md. Aliar Rahman conceived the study, developed the organic acid treatment protocols, performed the in vitro experiment and both broiler trials, collected the data, conducted the laboratory and statistical analysis, and wrote the original manuscript. Shathi Akter conducted the second broiler trial, gathered the experimental data, assisted in the blood biochemical analysis, and contributed to drafting the manuscript. Jesmin Aktar assisted with the second broiler trial, collected the data, did the feed analysis, and drafted the feed analysis methodology. Khan Md. Shaiful Islam supervised both broiler trials and the laboratory analysis, scientifically evaluated and interpreted the results, and examined the coherence among abstract, results, and conclusions. Mohammad Al-Mamun provided the resources for the organic acid treatments, supervised the broiler trials, scientifically contributed to the evaluation and interpretation of the data, and improved the drafting. Md. Abul Hashem performed the meat color analysis, contributed to the interpretation of the meat color data, assisted in refining the methodology, and strengthened the discussion related to meat color assessment. Rakhi Chowdhury developed the research objectives, conceived and designed the study, established the research protocols, secured grant funding, administered both projects by ensuring the availability of the necessary resources, supervised the entire research process, and drafted, edited, and substantially revised the manuscript. All authors critically reviewed the manuscript, approved its final version for publication, and agreed to be accountable for all aspects of the work.
